# Carbonization of single polyacrylonitrile chains in coordination nanospaces†

**DOI:** 10.1039/d0sc02048f

**Published:** 2020-06-05

**Authors:** Xiyuan Zhang, Takashi Kitao, Daniele Piga, Ryoto Hongu, Silvia Bracco, Angiolina Comotti, Piero Sozzani, Takashi Uemura

**Affiliations:** Department of Advanced Materials Science, Graduate School of Frontier Sciences, The University of Tokyo 5-1-5 Kashiwanoha, Kashiwa Chiba 277-8561 Japan t-uemura@k.u-tokyo.ac.jp; Department of Applied Chemistry, Graduate School of Engineering, The University of Tokyo 7-3-1 Hongo, Bunkyo-ku Tokyo 113-8656 Japan; Department of Material Science, University of Milano Bicocca Via R. Cozzi 55 20125 Milan Italy; Department of Synthetic Chemistry and Biological Chemistry, Graduate School of Engineering, Kyoto University Katsura, Nishikyo-ku Kyoto 615-8510 Japan; CREST, Japan Science and Technology Agency (JST) 4-1-8 Honcho, Kawaguchi Saitama 332-0012 Japan

## Abstract

It has been over half a century since polyacrylonitrile (PAN)-based carbon fibers were first developed. However, the mechanism of the carbonization reaction remains largely unknown. Structural evolution of PAN during the preoxidation reaction, a stabilization reaction, is one of the most complicated stages because many chemical reactions, including cyclization, dehydration, and cross-linking reactions, simultaneously take place. Here, we report the stabilization reaction of single PAN chains within the one-dimensional nanochannels of metal–organic frameworks (MOFs) to study an effect of interchain interactions on the stabilization process as well as the structure of the resulting ladder polymer (LP). The stabilization reaction of PAN within the MOFs could suppress the rapid generation of heat that initiates the self-catalyzed reaction and inevitably provokes many side-reactions and scission of PAN chains in the bulk state. Consequently, LP prepared within the MOFs had a more extended conjugated backbone than the bulk condition.

## Abstract 2

Accommodation of polyacrylonitrile in MOFs facilitated and regulated the transformation to ladder polymer in the carbonization process.

## Graphical abstract

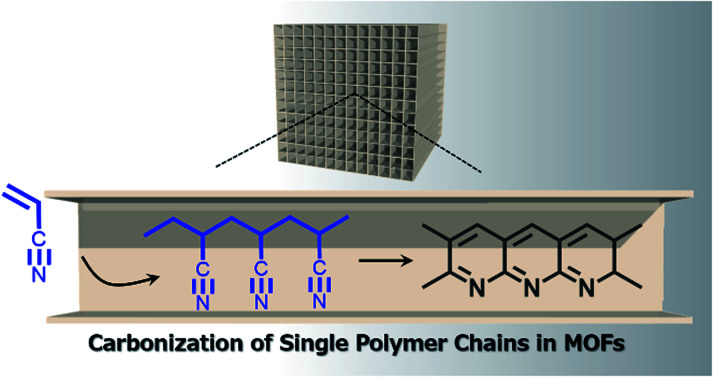

## Introduction

**Fig. 1:**
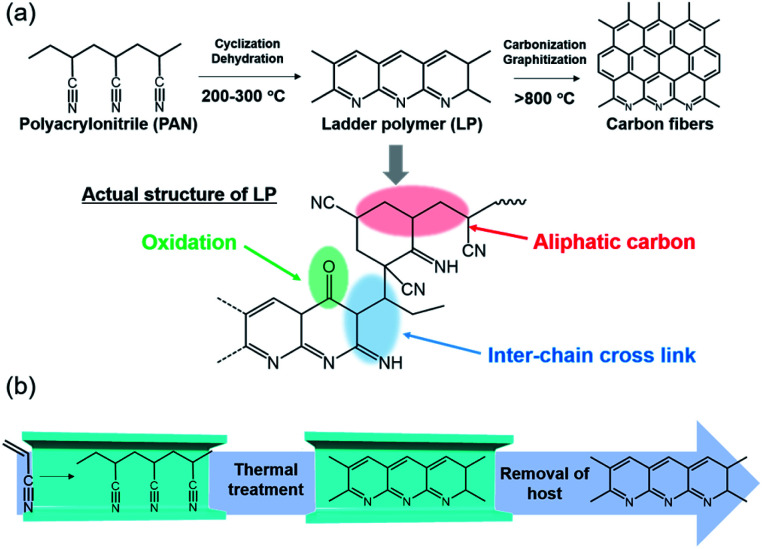
(a) Scheme of chemical reaction for carbon fibers and proposed structure of LP. (b) Schematic image for the stabilization reaction of single PAN chains inside the nanochannels of MOF.

Carbon fibers are extensively used in industrial fields because of their outstanding physical properties, low weight, and high chemical resistance.^1^ As the most vital precursor of carbon fibers, polyacrylonitrile (PAN) and its thermal transformation have been of great interest for many researchers in the past decades.^2–7^ Among several processes, stabilization is the most complicated and time-consuming step in the preparation of carbon fibers.^8,9^ The basic forms of stabilization reactions of PAN are illustrated in Fig. 1a, where PAN transforms to ladder polymer (LP) through intrachain cyclization and dehydration reactions. However, the actual structure is more complex and consists of cross-linking, aliphatic carbon, and carbonyl groups, as confirmed by a variety of characterization methods.^10–12^ The structural relationship between LP and resulting carbon fibers has been largely unexplored; hence, it is crucial to understand the mechanism of the stabilization process and control the structure of LP.^13,14^ However, in the bulk state, both intra- and interchain reactions proceed at the same time. Furthermore, the stabilization reaction is an exothermic process with the rapid evolution of heat, which provokes unfavorable side-reactions and scission of the polymer chains; thus, the process of stabilization is difficult to control in the bulk state.^15^

Confinement of polymer chains in porous materials is a feasible method to prevent the entanglement and conformational disorder occurring in bulk polymers, allowing regulation of their assembly structures.^16–19^ The stabilization reaction of PAN chains within well-defined nanospaces should provide insight into the effect of aggregation structures of PAN on their stabilization process and the structure of LP. For this purpose, we employed metal–organic frameworks (MOFs) prepared by self-assembly of metal ions and organic ligands, which offer a wide range of applications, such as gas storage and separation, catalysis, sensing, and drug delivery.^20–27^ The characteristic features of MOFs are their high regularity, robustness, and tunable channel structures at the molecular level. Taking advantage of these features, we can attain well-defined assemblies of polymer chains within the nanochannels, where the number, orientation, and environment of polymer chains are precisely controlled.^28–34^

Remarkable numbers of carbon materials have so far been fabricated by pyrolysis of MOFs themselves, in which micro- and nanometer-scale structural information of MOFs is transferred to the resulting carbon materials.^35,36^ The use of MOF channels as a nanosized reactor can offer opportunities to control structures of carbon materials at the molecular level, however, it has remained rare.^37^ Here, we encapsulated PAN in the one-dimensional (1-D) nanochannels of the MOFs by *in situ* polymerization, and subsequently performed the stabilization reaction (Fig. 1b). PAN was accommodated in the nanochannels of the MOFs in a single-chain manner, allowing investigation of how the interchain interactions affect their stabilization process and the structure of the LP. Note that rapid heat release, a fatal problem for the fabrication of carbon fibers, could be suppressed within the MOFs during the stabilization reaction, leading to the formation of LP with a more extended conjugated backbone than the bulk condition.

## Results and discussion

### Encapsulation of PAN into MOFs

**Fig. 2:**
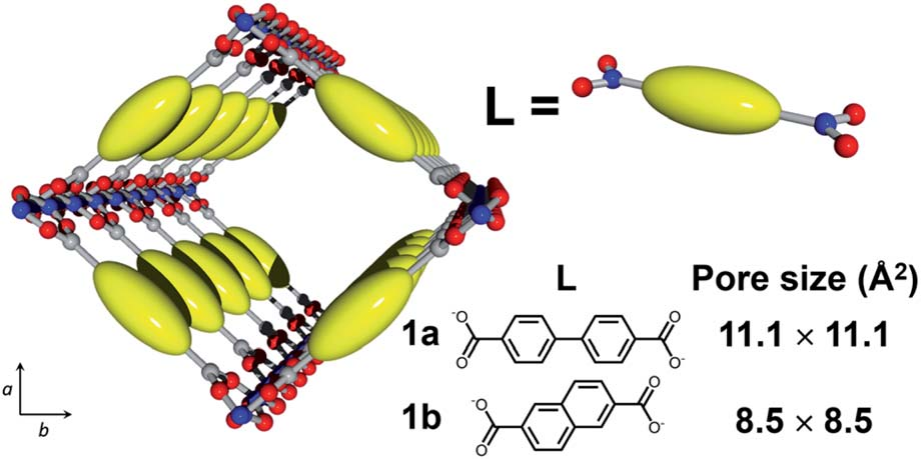
Schematic image for the crystal structure of **1** (Al, blue; O, red; C, gray; **1a**, L = 4,4′-biphenyldicarboxylate; **1b**, L = 2,6-naphthalenedicarboxylate).

Here, we employed MOF [Al(OH)(L)]_*n*_ (**1**; L = dicarboxylate; Fig. 2) with 1-D nanochannels as a host material because the precise control of pore size at the molecular level can be attained by changing the dicarboxylate ligands, L.^38^ In addition, [Al(OH)(L)]_*n*_ is highly tolerant of heat treatment at high temperature. In this work, we fabricated host–guest adducts using two MOFs with distinct channel sizes to investigate the effect of pore size on the stabilization reaction of single PAN chains. Considering the size of PAN, [Al(OH)(4,4′-biphenyldicarboxylate)]_*n*_ (**1a**, pore size = 11.1 × 11.1 Å^2^) and [Al(OH)(2,6-naphthalenedicarboxylate)]_*n*_ (**1b**, pore size = 8.5 × 8.5 Å^2^) can accommodate the PAN chains in a single-chain manner (Fig. 2 and S1†).

**Fig. 3:**
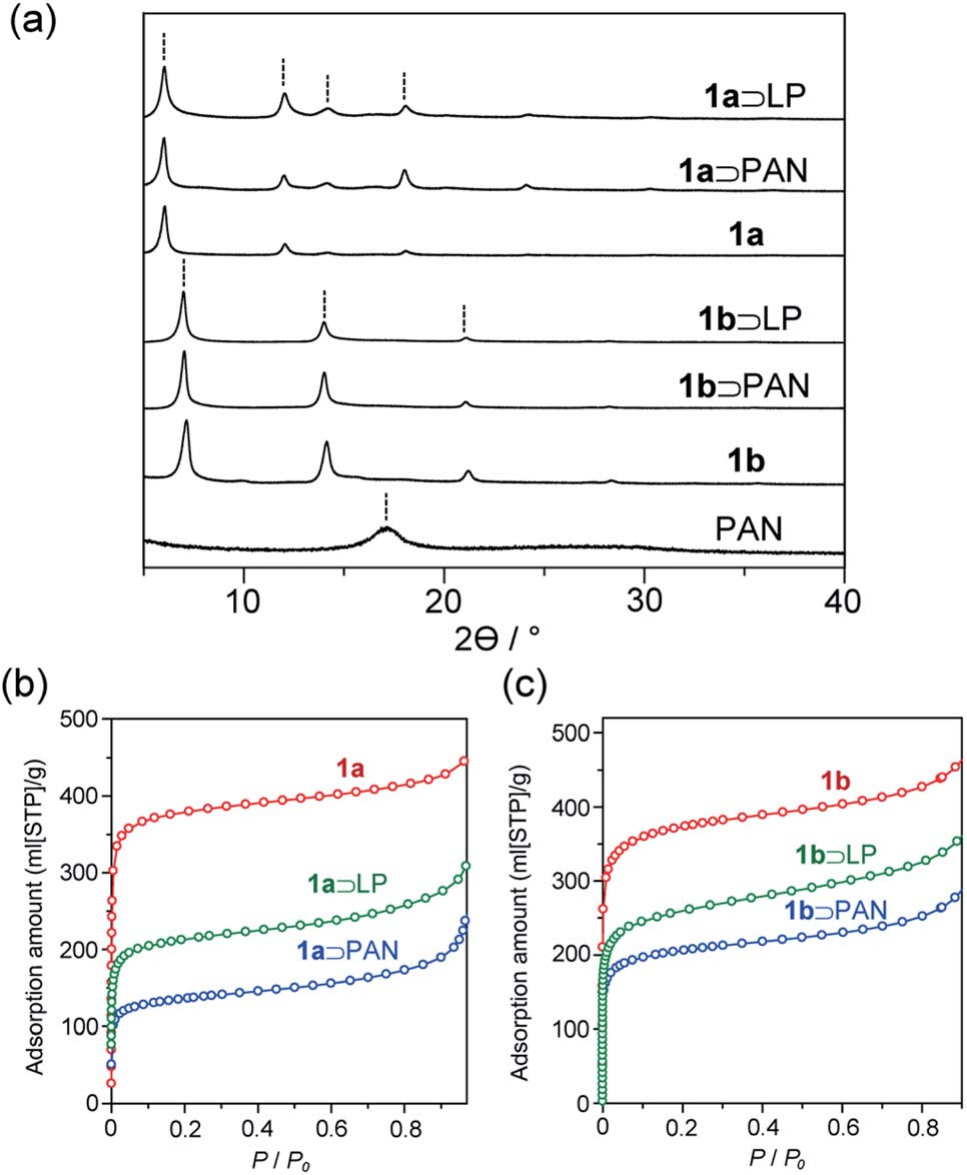
(a) PXRD patterns of PAN, **1**, **1**⊃PAN, and **1**⊃LP. (b and c) N_2_ adsorption isotherms of (b) **1a**, **1a**⊃PAN, **1a**⊃LP, (c) **1b**, **1b**⊃PAN, and **1b**⊃LP.

The activated MOFs were soaked in acrylonitrile monomer in the presence of azobisisobutyronitrile as an initiator followed by removal of excess monomer under reduced pressure to give the monomer adducts. Radical polymerization was then carried out at 100 °C for 24 h, affording **1** and PAN composites (**1**⊃PAN). Formation of the composites was confirmed by powder X-ray diffraction (PXRD). Despite its atactic stereoisomerism, PAN contains a partially crystalline region, where the helical polymer chains pack in a pseudo-hexagonal cell.^39^ The diffraction patterns of **1**⊃PAN did not contain a peak ascribable to crystalline PAN (Fig. 3a). No change in the peak positions, corresponding to **1**, was observed after the polymerization. Scanning electron microscopy (SEM) showed that the morphology of the particles of **1** remained unchanged during the polymerization (Fig. S2†). In addition, the N_2_ adsorption isotherms of **1**⊃PAN exhibited a decrease in the adsorption capacity compared with that of **1** (Fig. 3b and c). The overall characterizations of **1**⊃PAN confirmed that polymerization proceeded only inside the nanochannels with maintaining the framework structures.^40^

PAN could be thoroughly analyzed once it was liberated from the hosts using aqueous sodium ethylenediaminetetraacetate (NaEDTA) solution. ^1^H NMR and gel-permeation chromatography measurements presented the typical characteristics of PAN without any impurities (PAN from **1a**, *M*_n_ = 40 000, *M*_w_/*M*_n_ = 1.5; PAN from **1b**, *M*_n_ = 20 000, *M*_w_/*M*_n_ = 1.5; Fig. S3–S5†). The stereoisomerism of PAN was atactic regardless of the pore size of the hosts. Based on the mass of the recovered PAN, the molar ratios between the host ligand and the monomer unit of PAN were 1 : 1.2 and 1 : 1.0 for **1a**⊃PAN and **1b**⊃PAN, respectively. This result ensures that PAN does not exceed the amount necessary to fill the nanochannels.

### Stabilization reaction of PAN within **1**

The stabilization reaction of PAN proceeds around 280 °C in the bulk state, in which linear polymeric structure converts to an aromatic ladder structure. Since oxygen can facilitate the dehydration reaction along the backbone, resulting in the highly stabilized aromatic ladder structure,^10^**1**⊃PAN was thermally treated at 280 °C for 24 h in air. The color of the composites of **1** including PAN was drastically changed from white to dark brown during the heating treatment. Indeed, UV-vis spectra of **1**⊃LP exhibited red-shifted absorption extending into the visible region with respect to **1**⊃PAN (Fig. S6†). Because the absorption band of LP, corresponding to n–π* and π–π* transitions, lies in the visible light region, this suggested the formation of LP in **1** (**1**⊃LP).^41^ PXRD, SEM, and N_2_ adsorption measurements confirmed that the stabilization reaction proceeded within the hosts.^29,32^ The nanovessels retained their crystalline structure, as demonstrated by PXRD of **1**⊃LP (Fig. 3a). SEM showed that the morphology (size, shape, and surface) of the particles of **1** remained unchanged (Fig. S2†). The lower N_2_ adsorption capacity of **1**⊃LP than that of **1** alone was indicative of the encapsulation of LP in the nanochannels (Fig. 3b and c). However, the adsorption amounts of the composites slightly increased after the transformation to LP in **1**, which would be attributed to a decrease in occupied volumes of the guest polymers through the formation of planar ladder structures.

**Fig. 4:**
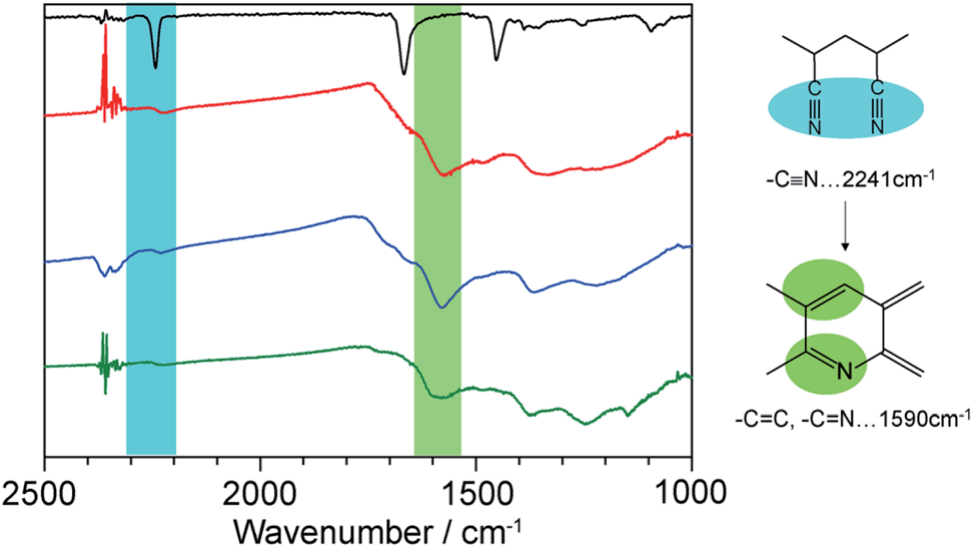
IR spectra of neat PAN (black), LP liberated from **1a** (red) and **1b** (blue), and LP prepared from neat PAN (green).

**1**⊃LP was then treated with an aqueous solution of NaEDTA to decompose the hosts, providing LP as a dark brown powder. The complete removal of the hosts was confirmed using SEM-energy dispersive X-ray analysis (Fig. S7†). We first characterized the chemical structures of LP using FT-IR spectroscopy measurements. The thermal stabilization process involves the transformation of the C≡N group into C=N by cyclization process and C=C unsaturation of the polymer main chain. The intensity of the C≡N absorption band at 2241 cm^−1^ decreased while a new peak of C=N and C=C stretching mode around 1590 cm^−1^, appeared (Fig. 4). This indicated that the cyclization and dehydration reactions took place within the nanochannels of **1** as is the case with the bulk condition.

The stabilization process of the neat PAN involves competitive thermal reactions, including not only cyclization and dehydration but also chain scission and cross-linking, leading to the formation of the complex structure (Fig. 1a).^10^

**Fig. 5:**
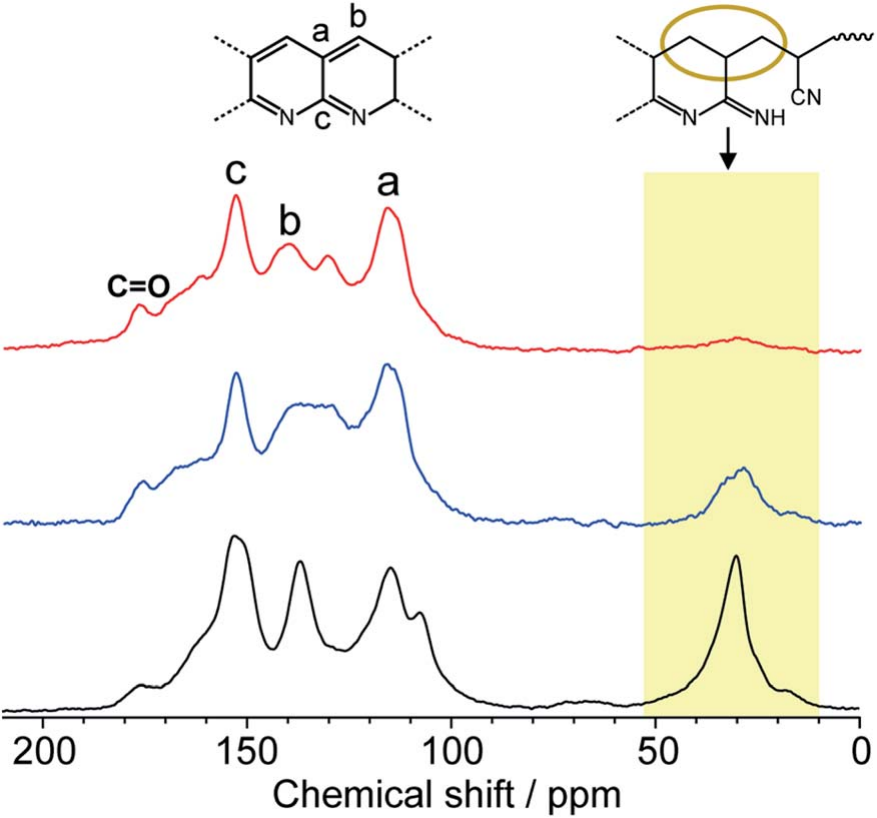
Solid-state ^13^C NMR spectra of LP prepared from neat PAN (black) and liberated from **1a** (red) and **1b** (blue).

**Fig. 6:**
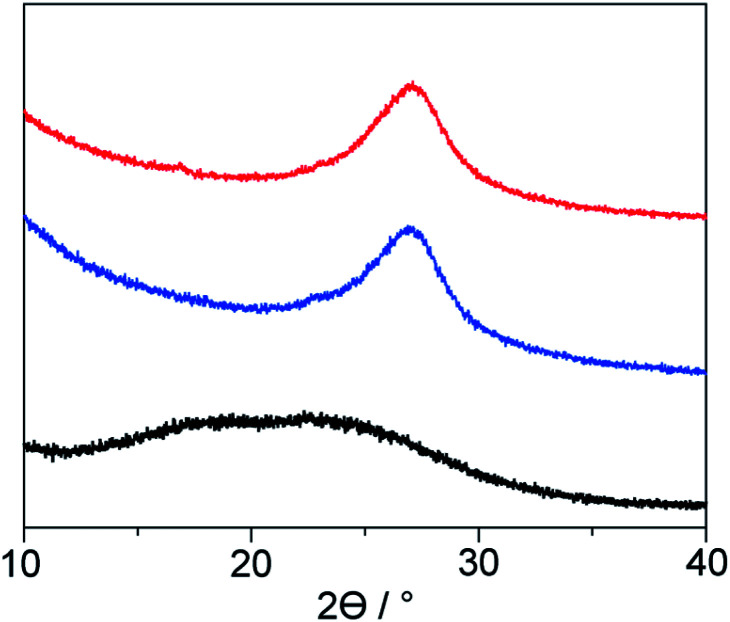
PXRD patterns of LP prepared from neat PAN (black) and in **1a** (red) and **1b** (blue).

Solid-state NMR is one of the most powerful techniques for detailed structural analysis, which allowed us to explore even the complex molecular structure of LP. In the case of LP prepared from neat PAN, formation of the aromatic ladder structure was demonstrated in ^13^C NMR by peaks around 100–160 ppm and the occurrence of oxidative reactions were detected by C=O carbon resonances at about 176 ppm (Fig. 5). However, the spectra revealed that LP had an incompletely stabilized structure, as shown by an intense peak around 29 ppm, assignable to aliphatic carbons (Fig. 5 and 1b). The recovered LP from **1** also showed peaks for aromatic carbons; whereas note that almost no peak for the aliphatic carbons was observed, which highly contrasted with the neat system. These obtained results were indicative of the formation of LP with a highly conjugated ladder structure within the nanochannels of **1**. This was supported by the analysis of their microstructures using PXRD measurements. The recovered LP from **1** had a rigid backbone and showed a clear diffraction peak around 27° 2theta, corresponding to the inter-chain stacking (Fig. 6).^42^ In contrast, LP synthesized under the neat condition presented a broad peak ranging from 10° to 30° 2theta, which could be attributed to the aliphatic backbone as well as cross-linking that hindered interchain stacking. Owing to the effective interchain packing, the density of LP from **1b** was determined to be 1.59 g cm^−3^ using helium pycnometry, which was indeed higher than that of LP prepared from neat PAN(1.54 g cm^−3^).

**Fig. 7:**
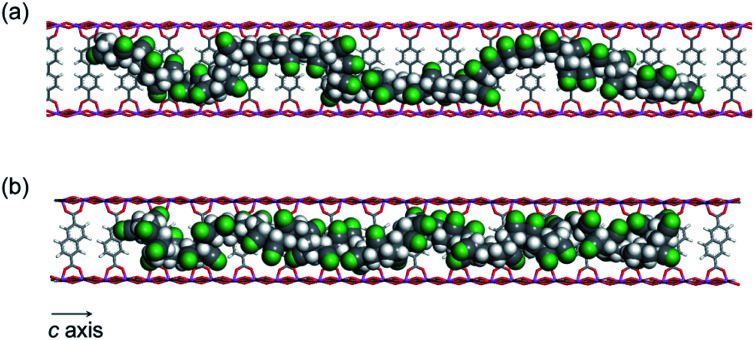
MD structures of the PAN chains confined within (a) **1a** and (b) **1b** viewed along the c-axis (Al, blue; O, red; C, gray; N, green; H, white).

While the thermal transformation of neat PAN involves incomplete stabilized structures with aliphatic carbons in a large quantity (Fig. 1a and 5), PAN confined in **1** dominantly provided LP with extended conjugated structures. During the thermal stabilization process, oxygen is known to facilitate the dehydration reaction of the PAN chains, which combine with cyclization between nitrile groups, leading to the formation of a conjugated ladder structure.^10,43^ However, in the bulk state, the PAN chains are closely packed with each other with partially crystalline domains, which makes it difficult for oxygen to access the polymer chains.^44^ To explore confinement effects of the MOFs on the stabilization reaction, we evaluated the configuration of the PAN chains within the nanochannels using molecular dynamics (MD) method (Fig. 7). The initial model structures of **1**⊃PAN were constructed, in which the molar ratios between the ligands of **1** and the monomer unit of PAN were set based on the actual loading amounts of PAN (**1a**⊃PAN, 1 : 1.2; **1b**⊃PAN, 1 : 1.0). The PAN chains were found to be dispersed within each nanochannel in a single-chain fashion. Despite the accommodation of PAN in the nanochannels, efficient access of oxygen molecules to the polymer chains could allow for the dehydration reaction because of significant void space around the PAN chains (Fig. 7), as was also supported by N_2_ adsorption results (Fig. 3b and c). Indeed, the small carbonyl peak at 176 ppm observed in the solid-state NMR spectra of LP (Fig. 5) clearly demonstrated access of oxygen to the polymer chains. Note that the amount of aliphatic carbons in LP slightly increased with a decrease in the pore size of **1** (Fig. 5), which was probably because of lower accessibility of oxygen as well as lower mobility of the PAN chains in the narrower channels.

**Fig. 8:**
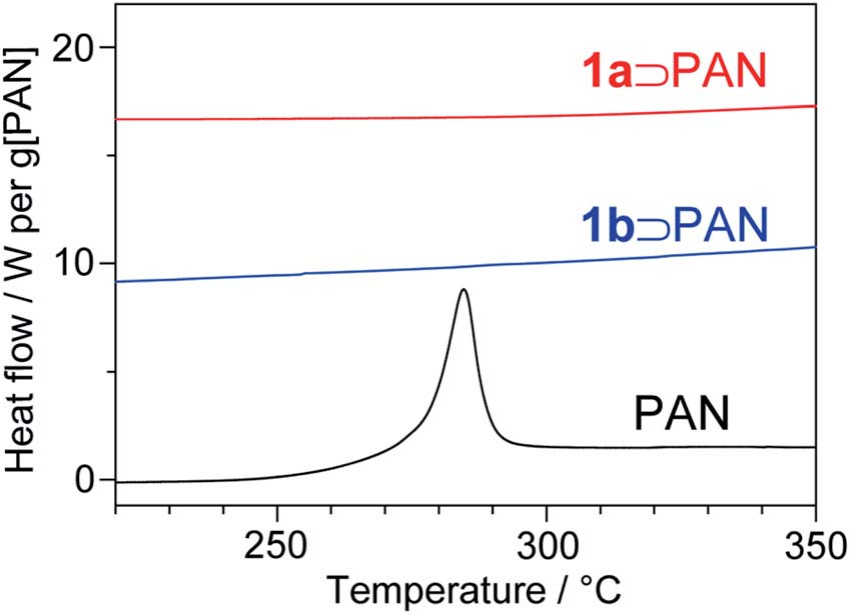
DSC heating curves of **1a**⊃PAN, **1b**⊃PAN, and neat PAN.

The stabilization reaction of PAN involves abrupt heat release, as shown by a sharp exothermic peak around 280 °C in the differential scanning calorimetry (DSC) curve for the neat PAN (Fig. 8). Rapid heat generation during the stabilization process is a serious problem for the fabrication of carbon fibers because it provokes unfavourable side-reactions and scission of the PAN chains.^45^ A striking result was obtained from a study of the thermal behaviour of PAN in **1**. Although full characterization of LP confirmed that the stabilization reaction proceeded within the nanochannels, no exothermic peak was detected in the DSC curve of **1**⊃PAN, which highly contrasted to the neat system (Fig. 8). There have been a few attempts to stabilize PAN chains confined in porous materials. Although the assembled structures of PAN within the pores were different from the structure in the bulk state, the resulting host–guest composites clearly showed exothermic peaks.^46,47^ To our knowledge, suppression of abrupt heat release has never been achieved during the stabilization reaction of PAN. MOFs have intrinsically low thermal conductivity because of their heterogeneity of atomic masses and stiffness of bonds.^48^ PAN chains were accommodated in each nanochannel in a single-chain fashion. Thus, we envisage a probable mechanism in which heat transfer between the polymer chains would be inhibited, suppressing the self-catalyzed reaction accompanied by abrupt heat release.

For the production process of carbon fibers, of particular interest is the issue of mitigating thermal runaways.^2^ To avoid thermal runaway, the stabilization process requires rigorous control of the heating rate and temperature. By utilizing the MOF channels as host matrices, we have succeeded in the suppression of rapid heat generation for the first time, which would provide fundamental insight into the stabilization reaction for future design and development of the manufacturing process for carbon fibers.

## Conclusion

Chemical reaction pathways of PAN during the stabilization reaction have been studied theoretically and experimentally over the past few decades. However, the reaction mechanism of the stabilization reaction has remained unclear because intra- and interchain reactions take place simultaneously in the bulk state. The characteristic feature of MOFs is their tunable surface functionality at the molecular level, which enables us to control the environments of polymer chains. In this work, we have demonstrated a facile methodology for the isolation of PAN chains in a single-chain manner using the MOFs as a host, allowing investigation of how the interchain interactions affect the stabilization reaction. Isolation of PAN chains in the nanochannels achieved suppression of rapid heat generation during the stabilization reaction. As a result, we obtained LP that had a more extended conjugated system with smaller amounts of aliphatic carbons than the bulk condition. We believe that these findings could provide further insight into the transformation reaction from LP to carbon fibers to enhance their properties. Because of the regulated and extended conjugated backbone with heteroatom doped structure, the formation of LP in the MOF nanochannels would represent unique electronic and magnetic properties. We expect that LP will provide opportunities for various applications, including opt-electronic nanodevices, batteries, catalysts, electrode materials with high energy storage density.^49–53^

## Conflicts of interest

There are no conflicts to declare.

## Supplementary Material

SC-011-D0SC02048F-s001
