# Supplementary material for: Carbonization of single polyacrylonitrile chains in coordination nanospaces
Source: Chem Sci. 2020 Jun 5;11(39):10844–9. doi: 10.1039/d0sc02048f (PMC8162375; doi:10.1039/d0sc02048f)
Supplement: SC-011-D0SC02048F-s001 [file SC-011-D0SC02048F-s001.pdf]

Electronic Supplementary Material (ESI) for Chemical Science.
This journal is © The Royal Society of Chemistry 2020

Supporting Information for

Carbonization of single polyacrylonitrile chains in coordination nanospaces

Xiyuan Zhang,[a] Takashi Kitao,[a,b] Daniele Piga,[c] Ryoto Hongu,[d] Silvia Bracco,[c] Angiolina Comotti,[c] Piero Sozzani,[c] Takashi Uemura*[a,b,e]

[a]Department of Advanced Materials Science, Graduate School of Frontier Sciences, The University of Tokyo, 5-1-5 Kashiwanoha, Kashiwa, Chiba 277-8561, Japan.

[b]Department of Applied Chemistry, Graduate School of Engineering, The University of Tokyo, 7-3-1 Hongo, Bunkyo-ku, Tokyo 113-8656, Japan.

[c]Department of Material Science, University of Milano Bicocca, Via R. Cozzi 55, 20125 Milan, Italy.

[d]Department of Synthetic Chemistry and Biological Chemistry, Graduate School of Engineering, Kyoto University, Katsura, Nishikyo-ku, Kyoto 615-8510, Japan.

[e]CREST, Japan Science and Technology Agency (JST), 4-1-8 Honcho, Kawaguchi, Saitama 332-0012, Japan.

Experimental

Materials

All reagents were purchased from commercial sources unless otherwise noted. Azobisisobutyronitrile (AIBN) was purified by recrystallization from ethanol, and acrylonitrile (AN) was purified by distillation under vacuum. [Al(OH)(4,4′-biphenyldicarboxylate)]$_n$ (**1a**; pore size = 11.1 × 11.1 Å^2) and [Al(OH)(2,6-naphthalenedicarboxylate)]$_n$ (**1b**; pore size = 8.5 × 8.5 Å^2) were prepared according to previously described methods.[1]

Stabilization reaction of polyacrylonitrile (PAN) in 1

Host compound **1** (200 mg) was activated by evacuation (0.3 kPa) at 130 °C overnight to remove residual solvent within the pore. Then, **1** was immersed in AN (1 mL) and AIBN (4 mg) mixture at room temperature to incorporate AN and AIBN into the pores. Excess AN was removed under reduced pressure (0.3 kPa). Subsequently, radical polymerization was performed at 100 °C for 24 h under N_2 atmosphere, affording the composite between **1** and PAN (**1**⊃PAN). **1**⊃PAN was collected after washing with MeOH and dried under reduced pressure. **1**⊃PAN underwent thermal treatment at 280 °C for 24 h under air to perform the stabilization reaction of PAN within **1**, providing **1** and ladder polymer (LP) composite (**1**⊃LP).

Liberation of PAN and LP from 1

To isolate the polymers accommodated in **1**, the resulting composites were stirred in 0.05 M sodium ethylenediaminetetraacetate aqueous solution for 24 h to decompose the framework of **1**. The collected materials were washed with water and dried under reduced pressure at room temperature.

Physical measurements

Powder X-ray di□raction (PXRD) data were collected on a Rigaku SmartLab Diffractometer using a Cu anode and a Kα monochromator (λ = 0.154 nm). ^{13}C solid-state NMR experiments were carried out with a Bruker AVANCE 300 instrument operating at a static field of 7.04 T equipped with high-power amplifiers (1 kW) and a 4 mm double resonance magic-angle spinning (MAS) probe. $^{13}C\{^{1}H\}$ ramped-amplitude Cross Polarization MAS experiments were performed at room temperature at a spinning speed of 12.5 kHz using a contact time of 2 and 0.05 ms with a recycle delay of 5 s. The 90° pulse for proton was 2.9 μs. The adsorption isotherms of N_2 at 77 K were measured using BELSORP-mini equipment. Before the adsorption measurements, the sample was treated under reduced pressure (<10^{-2} Pa) at 373 K for 5 h. Scanning electron microscopy-energy dispersive X-ray (SEM-EDX) measurements were conducted using a HORIBA EMAXEvolution EX-370 attached to a HITACHI S-3000N operated at an accelerating voltage of 20 kV. Samples were placed on a conducting carbon tape attached by the SEM grid and then coated with platinum. IR spectra were measured using a JASCO FT/IR-4200. Differential scanning calorimetry (DSC) was carried out with Seiko Instruments DSC 6220 under N_2 atmosphere. The NMR spectra were obtained using a JEOL A-500 spectrometer operating at 500 MHz. The gel-permeation chromatography (GPC) measurements were performed in DMF at 40 °C on three linear-type polystyrene gel columns (Shodex K-805 L) that were connected to a JASCO PU-980 precision pump, a JASCO RI-930 refractive index detector, and a JASCO UV–970 UV–vis detector set at 256 nm. UV–vis spectra were recorded using a JASCO V-670 spectrophotometer. The Helium Pycnometer analysis was performed with Micromeritics AccuPyc II 1340 Pycnometer with a 1 cm^3 sample holder.

Molecular dynamics (MD) simulations

MD simulations were performed using the Materials Studio Modeling v4.4 software package (Accelrys Inc., San Diego, CA, USA) using the Universal Force Field, as implemented in the Forcite module. The charges were dealt with by the charge equilibration method in this system. The initial structure of **1** was generated based on the X-ray crystal structure of **1**. The quench dynamics with the optimized structures were conducted at 493 K, and then, MD calculations were carried out at 298 K for 1000 ps under NVE conditions.

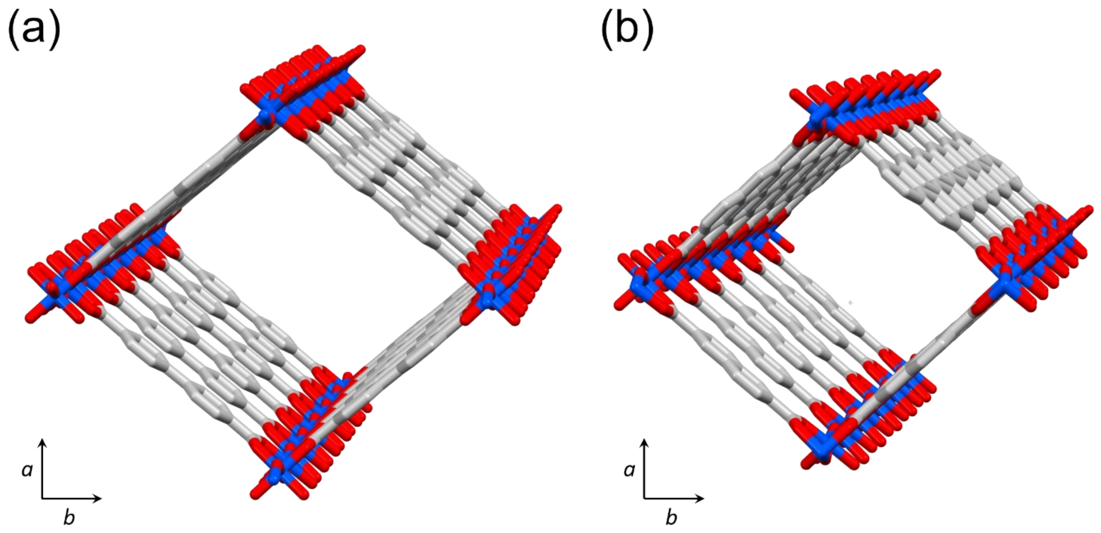

Fig. S1 Crystal structures of (a) **1a** and (b) **1b** (Al, blue; O, red; C, gray). Hydrogen atoms are omitted for clarity.

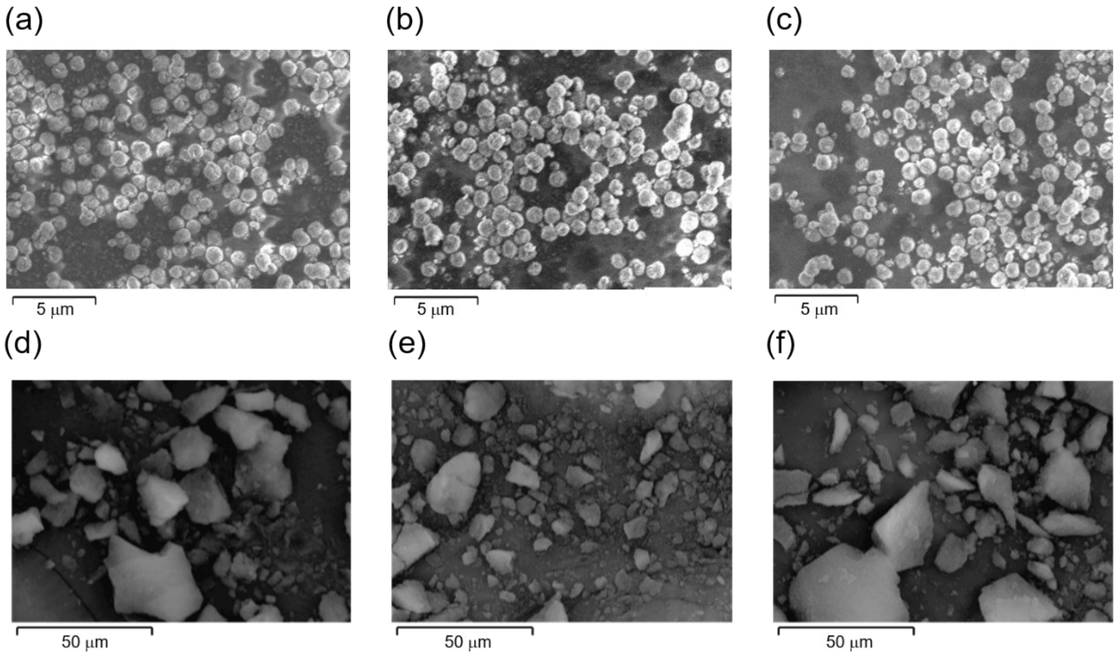

Fig. S2 SEM images of (a) **1a**, (b) **1a**⊃PAN, (c) **1a**⊃LP, (d) **1b**, (e) **1b**⊃PAN, and (f) **1b**⊃LP. The size and morphology of the host crystals were almost the same as those of the composites, showing that polymerization of AN and the stabilization reaction proceeded only within the pores of **1**.

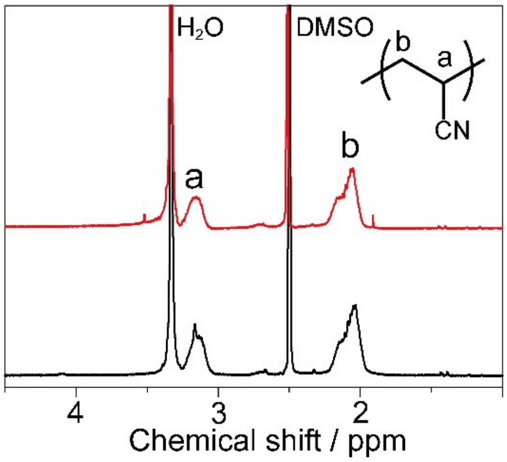

Fig. S3 ^{1}H NMR spectra of PAN liberated from **1a** (red) and **1b** (black) in DMSO-d$_6$.

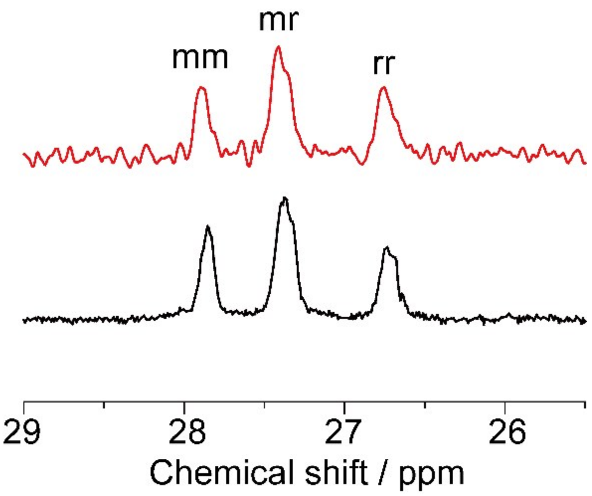

Fig. S4 ^{13}C NMR spectra of PAN liberated from **1a** (red) and **1b** (black) in DMSO-d$_6$, in which the ratios of mm:mr:rr were calculated to be 23:50:27 and 27:47:26, respectively.

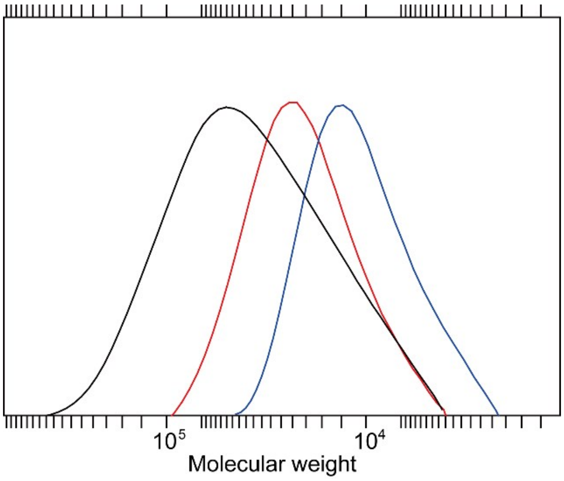

Fig. S5 GPC profiles of PAN liberated from **1a** (red), **1b** (blue), and bulk PAN synthesized by solution polymerization (black) (PAN from **1a**, M_n = 40,000, M_w/M_n = 1.5; PAN from **1b**, M_n = 20,000, M_w/M_n = 1.5, bulk PAN, M_n = 84,000, M_w/M_n = 1.9). In general, mobility of monomers is restricted in nanoconfined polymerization systems, which often lowers the efficiency of chain propagation. Therefore, molecular weights of PANs produced in **1** were smaller than that prepared by free radical polymerization. As the nanochannels narrowed, the molecular weight of PAN was found to decrease probably because of the strong confinement effect.[2] However, the chain length of PAN synthesized in **1** was long enough (M_n > 20,000) to show the properties identical to the bulk-synthesized PAN.

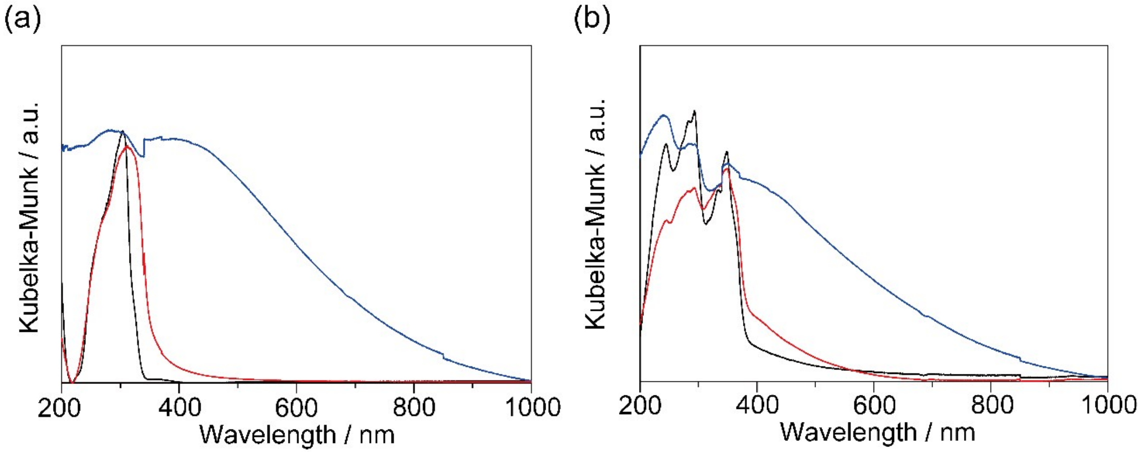

Fig. S6 Solid-state UV–vis spectra of (a) **1a** (black), **1a**⊃PAN (red), **1a**⊃LP (blue), (b) **1b** (black), **1b**⊃PAN (red), and **1b**⊃LP (blue). UV–vis spectra of **1**⊃LP showed red-shifted absorption extending into the visible region with respect to **1**⊃PAN, suggesting formation of LP within **1**.[3]

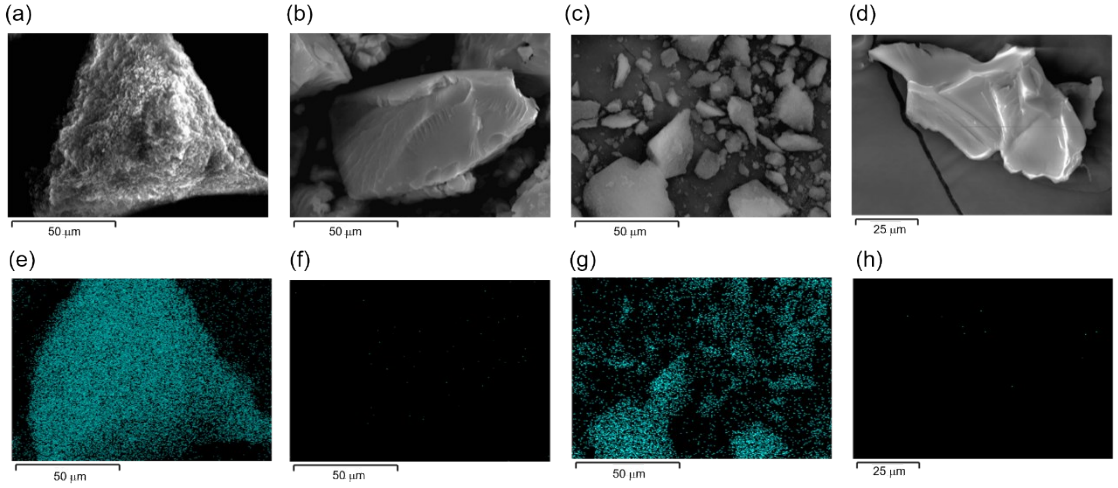

Fig. S7 SEM images of (a, b) **1a**⊃LP and **1b**⊃LP, (c, d) LP liberated from **1a** and **1b**, respectively. EDX analysis (Al mapping) of the corresponding (e, f) **1a**⊃LP and **1b**⊃LP, (g, h) LP liberated from **1a** and **1b**, respectively, which demonstrated the removal of **1** during the polymer recovery process.

REFERENCES

1. I. Senkovska, F. Hoffmann, M. Fröba, J. Getzschmann, W. Böhlmann and S. Kaskel, *Microporous Mesoporous Mater.*, 2009, **122**, 93−98.
2. J. Hwang, H. C. Lee, M. Antonietti and B. V. K. J. Schmidt, *Polym. Chem.*, 2017, **8**, 6204−6208.
3. T. Sun, Y. Hou and H. Wang, *J. Appl. Polym. Sci.*, 2010, **118**, 462−468.
